# Phytochemical Profiling and Anti-Fibrotic Activities of the Gemmotherapy Bud Extract of *Corylus avellana* in a Model of Liver Fibrosis on Diabetic Mice

**DOI:** 10.3390/biomedicines11061771

**Published:** 2023-06-20

**Authors:** Cornel Balta, Hildegard Herman, Alina Ciceu, Bianca Mladin, Marcel Rosu, Alciona Sasu, Victor Eduard Peteu, Sorina Nicoleta Voicu, Mihaela Balas, Mihaela Gherghiceanu, Anca Dinischiotu, Neli Kinga Olah, Anca Hermenean

**Affiliations:** 1 “Aurel Ardelean” Institute of Life Sciences, Vasile Goldis Western University, 86 Rebreanu, 310414 Arad, Romania; balta.cornel@uvvg.ro (C.B.); herman.hildegard@uvvg.ro (H.H.); alina.ciceu@uvvg.ro (A.C.); mladin.bianca@uvvg.ro (B.M.); rosu.marcel@uvvg.ro (M.R.); sasu.alciona@uvvg.ro (A.S.); hermenean.anca@uvvg.ro (A.H.); 2 Victor Babes National Institute of Pathology, 050096 Bucharest, Romania; peteuvictoreduard@gmail.com (V.E.P.); mihaela.gherghiceanu@ivb.ro (M.G.); 3 Department of Biochemistry and Molecular Biology, Faculty of Biology, University of Bucharest, 050095 Bucharest, Romania; sorina.voicu@bio.unibuc.ro (S.N.V.); mihaela.balas@bio.unibuc.ro (M.B.); anca.dinischiotu@bio.unibuc.ro (A.D.); 4 Department of Cellular and Molecular Biology and Histology, Faculty of Medicine, Carol Davila University of Medicine and Pharmacy, 050474 Bucharest, Romania; 5 Faculty of Pharmacy, Vasile Goldis Western University, 86 Rebreanu, 310414 Arad, Romania; 6 Faculty of Medicine, Vasile Goldis Western University, 86 Rebreanu, 310414 Arad, Romania

**Keywords:** liver fibrosis, gemmotherapy, *Corylus avellana*, TGF, MMP, oxidative stress

## Abstract

In this study, we aimed to explore the hepatoprotective effects of the gemmotherapy bud extract of *Corylus avellana* in a model of liver fibrosis on diabetic mice. An evaluation of total flavonoids and polyphenols contents and LC/MS analyses were performed. Experimental fibrosis was induced with CCl_4_ (2 mL/kg by i.p. injections twice a week for 7 weeks) in streptozotocin-induced diabetic mice. Our results showed a content of 6–7% flavonoids, while hyperoside and chlorogenic acids were highlighted in the bud extract. Toxic administration of CCl_4_ increased oxidative stress, mRNA expression of the transforming growth factor-β1 (*TGF-β1*) and *Smad 2/3*, and reduced *Smad 7* expression. Furthermore, up-regulation of α-smooth muscle actin (*α-SMA*) revealed an activation of hepatic stellate cells (HSCs), while collagen I (*Col I*) up-regulation and matrix metalloproteinases (*MMPs*) unbalance led to an altered extracellular matrix enriched in collagen, confirmed as well by a trichrome stain and electron microscopy analysis. Treatment with gemmotherapy extract significantly restored the liver architecture and the antioxidant balance, and significantly decreased collagen deposits in the liver and improved the liver function. Our results suggest that *Corylus avellana* gemmotherapy extract may have anti-fibrotic effects and could be useful in the prevention and treatment of liver fibrosis. The hepatoprotective mechanism is based on HSC inhibition, a reduction in oxidative stress and liver damage, a downregulation of the TGF-β1/Smad signaling pathway and a MMPs/TIMP rebalance.

## 1. Introduction

Liver fibrosis is the excessive accumulation of fibrous connective tissue in the liver. It can be caused by different factors, including viruses (such as hepatitis B or C), alcohol, and nonalcoholic fatty liver disease [1].

The molecular mechanisms underlying liver fibrosis involve a complex interplay between various signaling pathways and cell types within the liver. One key component of the molecular mechanism is the activation of hepatic stellate cells (HSCs). HSCs are a type of cell that normally stores fat in the liver. However, when the liver is damaged, HSCs become activated and start promoting fibrogenic molecules that encourage fibroblasts and bone marrow-derived myofibroblasts to secrete collagen and thereby induce fibrosis [2,3].

Other key players in the molecular mechanism of liver fibrosis include inflammatory cells and growth factors, the transforming growth factor-beta (TGF-β1) and the platelet-derived growth factor (PDGF) [4]. These molecules can stimulate the activation of HSCs and the production of collagen, leading to the development of fibrosis. Additionally, the molecular mechanism of liver fibrosis involves the regulation of extracellular matrix (ECM) remodeling, which is the process by which the ECM is broken down and replaced with a new tissue [5]. In healthy livers, ECM remodeling is tightly regulated, but in cases of liver fibrosis, this process becomes dysregulated, leading to the accumulation of excess collagen and other ECM proteins [6].

*Corylus avellana*, known as hazelnut, is a deciduous tree that is native to Europe and parts of Asia. Its hard shell contains kernels, with a high content of healthy lipids. Phenolics are the most abundant, while taxane derivatives are isolated from leaves [7]. Components of *Corylus avellana*, such as hazelnuts, indoleacetic acid glycosides and indole alkaloids, have been shown to have antimicrobial, anti-inflammatory and antioxidant activities [8]. *C. avellana* extract has an immune adjuvant effect, by enhancing the human macrophage response to *Staphylococcus aureus* due to the upregulation of the anti-inflammatory and iron metabolism genes [9]. However, hazelnut extract has some beneficial effects on carbon tetrachloride (CCl_4_) toxicosis, by reducing hepatocytolysis as well as histological lesions and returning the activity of some hepatic enzymes to normal values [10]. Moreover, the nutraceutical potential of *Corylus avellana* daily supplements for obesity and related dysmetabolism was assessed in a mouse model of a metabolic syndrome (a high-fat diet) [11].

Gemmotherapy is a form of herbal medicine that uses extracts made from the young shoots, or buds, of various plants. These extracts, known as gemmotherapy remedies, are thought to contain a high concentration of plant hormones and growth factors that can stimulate the healing process. On the subject of the gemmotherapy extract of *Corylus avellana,* we learned from traditional Chinese medicine (TCM) that hazel harmonizes the movement of Qi and fluids in the lungs and liver. However, it is important to note that the potential liver-protective effects of gemmotherapy remedies made from *Corylus avellana* buds have not been studied, and research is needed to fully understand their mechanisms of action and to determine their therapeutic effects and safety in this context.

With these perspectives, we aimed to evaluate for the first time the phytochemical composition of the gemmotherapy bud extract of *Corylus avellana*. Secondly, we examined in vivo the possible hepatoprotective and anti-fibrotic effects in a mouse model of liver fibrosis.

## 2. Materials and Methods

### 2.1. The Vegetal Raw Material

The *Corylus avellana* L. (hazelnut) fresh buds were harvested from forests situated near Cluj-Napoca, Romania, distant from urban areas and 5 km from any roads. For this study, the vegetal materials were harvested twice for two consecutive years, in February–March 2019 in January–February 2020, respectively. The buds were harvested at their maximum development, but before they opened and formed the young leaves. The vegetal material was identified in the QC laboratory of SC PlantExtrakt SRL. A voucher specimen was retained at each harvest. There were collected vegetal raw materials at the beginning and at the end of the harvesting period.

The harvesting was performed according to the EcoInspect Ro-008 certificate, respecting good collecting practices from wild flora, and preserving the biodiversity of the area.

### 2.2. The Preparation of Gemmotherapy Extracts

Hazelnut bud extract was obtained according to the French Pharmacopoeia and the European Pharmacopoeia, monograph 2.1.3, using a mixture of glycerin and 96% vol. ethyl alcohol (1:1) as a solvent. The plant was processed freshly. The extraction ratio was 1:20 dry part of the plant-solvent. The amount of solvent added was calculated according to the moisture content of the plant, which was 43–47%. The extraction was carried out at a cold temperature, by maceration, for 20 days, with daily mixing 2 × 10 min. After 20 days of mixing, the liquid was decanted from the plant material, and the rest of the plant was subjected to pressing at 350–400 atm, the pressed liquid being mixed with the decanted liquid. The extractive solution thus obtained is the *Corylus avellana* gemmotherapy extract. There were prepared extracts from each harvested batch. The yield of extraction was 86–97%.

### 2.3. The Evaluation of Total Flavonoids and Polyphenols Contents

The spectrophotometric quantification of total flavonoid and polyphenols was performed with a Cintra 101 spectrophotometer (Dunbar Road, Cayce, SC, USA). The method was adapted from the Romanian Pharmacopoeia, IXth edition. Briefly, 0.1 mL of gemmotherapy extract was mixed with 0.5 mL of phosphotungstic reagent, then completed to 25 mL with 15% sodium carbonate. Methanolic solutions of caffeic acid with concentrations of 12, 20, 28 and 40 μg/mL, respectively, were used as a standard. Each sample was processed and analyzed in triplicate and the results are the arithmetic mean of the individual determinations.

For total flavonoids quantification, the aluminium chloride solution and quercetin was used as a standard. The calibration curve was performed using different concentrations of quercetin solution (2.24; 6.72; 11.20; 15.68 and 22.40 μg/mL).

### 2.4. The LC/MS Analyses

The LC/MS analyses were performed on a Shimadzu Nexera I LC/MS-8045 (Kyoto, Japan) UHPLC system. The separation was carried out on a Luna C18 reversed-phase column (Torrance, CA, USA). The mobile phase was a gradient made from methanol (Merck, Darmstadt, Germany) and ultrapurified water (Merck Millipore, Billerica, MA, USA). Formic acid was used as an organic modifier.

The detection was performed on a quadrupole rod mass spectrometer operated with electrospray ionization (ESI), both in negative and positive MRM (multiple reaction monitoring) ion mode. Chlorogenic acid, gallic acid, salicylic acid, catechin, apigenin, chrysin, hyperoside, luteolin-*7-O*-glucosid, naringenin, quercetin and rutoside were used as standards. A quantity of 1 mL was injected from each standard at each concentration. The identification was performed by comparison of the MS. The identification and quantification were made based on the main transition from the MS spectra of the substance.

### 2.5. Animals and Experimental Design

The experimental protocol was approved by the Research Ethics Committee of the ”Vasile Goldis” Western University of Arad (152/02.10.2019). In order to induce diabetes, CD1 male adult mice were injected intraperitoneally (ip) with streptozotocin (STZ) solubilized in citrate buffer (STZ; 102 mg/kg). Blood glucose levels were measured after 4 h of fasting using a glucometer. Two weeks after STZ administration, mice with a blood glucose level higher than 200 mg/dL for two consecutive weeks were included in the study. Diabetic mice were then ip-injected with CCl_4_ (20% *v*/*v*, 2 mL/kg dissolved in olive oil) twice a week for 7 weeks and euthanatized after the last injection to confirm liver fibrosis (group 2—*DF group*). Spontaneous resolution of liver fibrosis was investigated in CCl_4_-treated diabetes animals after 2 weeks of self-recovery (group 3—*DFR*). In order to evaluate the antifibrotic effect of the *Corylus avellana* gemmotherapy extract, treatment was started at the end of 7 weeks of CCl_4_ treatment (6.711 μg flavonoid/kg body weight), using daily gavage administration for 14 consecutive days (group 4—*DCA group*). The dose was established by extrapolating the data collected from the doctors’ medical practice. The group 1 (*control*) received only the saline solution.

### 2.6. Biochemistry

The serum level of alkaline phosphatase (ALP) and aspartate aminotransferase (AST) was determined from cardiac blood samples using a biochemical analyzer (Mindray BS-120, Mindray Bio-Medical Electronics, Shenzhen, China).

### 2.7. Histology and Immunohistochemistry

Liver samples were fixed in a 4% paraformaldehyde solution, embedded in paraffin, and stained with a Gomori’s trichrome stain kit (38016SS1, Leica, Westford, MA, USA). Morphological analysis was performed using an Olympus BX43 microscope.

For immunohistochemistry, deparaffinized sections were incubated overnight at 4 °C with the primary antibodies TGF-β1 (sc-146), Smad 2/3 (sc-8332) and α-SMA (ab32575) using a 1:200 dilution. A Novolink Max Polymer (Leica Biosystems, Wetzlar, Germany) and 3,30-diaminobenzidine (DAB) were used for immunodetection. The nuclei were counterstained with hematoxylin. Images were assessed by light microscopy (Olympus BX43, Hamburg, Germany).

### 2.8. Electron Microscopy

The prefixation was performed in 2.7% glutaraldehyde solution in 0.1 M phosphate buffer, then washed in 0.15 M phosphate buffer (pH 7.2) and postfixated in 2% osmic acid solution in 0.15 M phosphate buffer. The samples were then dehydrated in acetone, followed by embedding in Epon 812 epoxy resin. The liver thin sections of 70 nm thickness were double-stained with solutions of uranyl acetate and lead citrate. The sections were analyzed using a TEM microscope (Morgagni268, FEI, Eindhoven, The Netherlands) at 80 kV. Data acquisition was performed with a MegaView III CCD using iTEM SIS software (Olympus Soft Imaging Software, version 3.0, Münster, Germany).

### 2.9. Quantitative Real-Time PCR Analysis

A real-time quantitative polymerase chain reaction (qPCR) was applied to assess mRNA expression of *TGF-β1*, *Smad 2*, *3*, *7*, *Col I*, *TIMP-1*, *α-SMA*, *MMP-1*, *MMP-2*, *MMP-3* and *MMP-9*. The total RNA extraction was performed with a Promega kit and quantification by spectrophotometry (NanoDropOne, Thermo Scientific, Waltham, MA, USA). The reverse transcription was performed in triplicate using a first strand cDNA synthesis kit (Thermo Scientific, Waltham, MA, USA) and the RT–PCR reaction with a Maxima SYBR Green master mix (Life Technologies, Carlsbad, CA, USA). The primers are shown in Table 1. Glyceraldehyde 3-phosphate dehydrogenase (GAPDH) was used as a reference gene under the same experimental protocol. Relative expression changes were determined using the 2 ^∆∆C(T)^ method [12].

### 2.10. Antioxidant Activity

#### 2.10.1. Preparation of Tissue Lysate

The total tissue lysate was obtained by homogenization, using an ultrasonicator (UP50H from Hielscher Ultrasound Technology, Teltow, Germany) at 100% amplitude in 0.1 M Tris-EDTA buffer solution, pH 7.4, at a ratio of 1 g tissue/10 mL. Proteins in tissue extracts were determined by the Lowry method (Lowry, 1951) [13] using BSA (bovine serum albumin) as a standard.

#### 2.10.2. Lipid Peroxidation Assay

The malondialdehyde (MDA) level was assessed using the method described by Del Rio [14]. The fluorescence measurement was performed in a Jasco FP-750 fluorimeter (Excitation 520 nm/Emission 549 nm) and the results were expressed in nmoles MDA/mg protein.

#### 2.10.3. Reduced Glutathione (GSH) Assay

GSH concentration was determined using a colorimetric detection kit (Arbor Assays Detectx™, Sigma-Aldrich, St. Louis, MO, USA). The proteins were removed by precipitation with 10% sulfosalicylic acid (SSA). The results were reported to the protein concentration of samples and expressed in nmoles GSH/mg protein.

#### 2.10.4. Advanced Oxidation Products of Proteins (AOPP)

The level of advanced oxidation products of proteins (AOPP) was determined spectrophotometrically according to the method described by Witko [15], using a standard curve based on chloramine T 100 µM. The concentration of AOPP expressed in units of chloramine was related to the protein concentration.

#### 2.10.5. Western Blotting Analysis

Protein expression of the nuclear factor erythroid 2-related factor 2 (Nrf-2), heme oxygenase-1 (HO-1), MMP-2 and MMP-9 were evaluated by the Western blotting technique. A quantity of 50 µg protein was loaded on gels and 10% of sodium dodecyl sulfate-polyacrylamide gel electrophoresis (SDS-PAGE) was performed. The gels were then transferred onto a polyvinylidene fluoride membrane (Immobilon-PVDF membrane, Merck, Darmstadt, Germany) in the presence of tris/glycine/methanol transfer buffer, at 350 mA, for 1.5 h using the wet transfer system. After blocking, the membranes were incubated overnight with 1:250 diluted primary antibodies anti-Nrf2 (Santa Cruz, Biotechnology, Dallas, TX, USA) and HO-1 (GeneTex, Inc., Irvine, CA, USA), 1:500 anti-MMP-2 (Santa Cruz, Biotechnology, Dallas, TX, USA), and anti-MMP-9 (Santa Cruz, Biotechnology, Dallas, TX, USA), as well as 1:1000 anti-β-actin (Santa Cruz, Biotechnology, Dallas, TX, USA). The membranes were then incubated for 1 h with anti-mouse, anti-rabbit, or anti-goat secondary antibodies conjugated with alkaline phosphatase and 5-bromo-4-chloro-3-indolyl-phosphate/nitro-blue tetrazolium (BCIP/NBT) was used as a chromogenic substrate. The membrane blots were visualized with a ChemiDoc imaging system (Bio-Rad, Hercules, CA, USA), and protein bands were quantified with Image Lab software (version 5.2, Bio-Rad, Hercules, CA, USA), and normalized to the β-actin.

#### 2.10.6. Enzymatic Activity of Metalloproteinases MMP-2 and MMP-9

The gelatin zymography method was used to measure the enzymatic activity of MMPs. An amount of 80 µg proteins was separated on a 10% SDS-PAGE containing 0.2 % gelatin, at 4 °C. The gels were then washed and incubated twice for 15 min with 50 mM of Tris HCl pH 7.6 renaturation buffer with 2.5% Triton X-100. Further, the gels were incubated overnight at 37 °C with 50 mM of Tris HCl pH 7.6 buffer containing 10 mM of CaCl_2_, 50 mM of NaCl, and 0.05% Brij 35 and stained with Coomassie brilliant blue solution for 1 h at room temperature. The de-staining of the gels was done with a solution of 10% methanol and 10% acetic acid in distilled water, twice for 30 min. The gels were analyzed using the ChemiDoc Imaging System (BioRad), and bands were quantified with Image Lab software (BioRad).

### 2.11. Statistical Analysis

The data were analysed with a GraphPad Prism 9.4.0 and expressed as a mean ± SD. Statistical analyses were performed using one-way ANOVA with the Bonferroni correction. A *p*-value of <0.05 was considered significant.

## 3. Results

### 3.1. Characterization of Corylus avellana Gemmotherapy Buds Extract

In Table 2 are presented the results obtained for the total flavonoids and total polyphenol content.

A high content of polyphenols was determined, of which 6–7% were flavonoids. Total polyphenols are in higher concentration in the earlier stage of the bud’s development and decrease during their growth, while the flavonoid increases. Depending on these contents, the optimal time to collect the buds can be established to be mid-February, when the buds are more developed. Even allowing for the fact that these determinations are quantitative and universally used, the flaws in these methods allow us to use these values only for guidance, the most precise analytical information regarding the individual flavonoids and other polyphenols being obtained by LC/MS analysis.

The LC/MS chromatograms of the studied gemmotherapy extracts and the identified compounds’ MS spectra are presented as Supplementary Data (Figures S1–S10).

In Table 3 are presented the results of quantitative determinations by LC/MS.

The results showed that the main polyphenol is a flavonoid, hyperoside, that has a sensitive constant concentration in the extract, independent of the development period or season. The same results can be observed in another quercetin derivative, rutoside, which is at a level of ¼ of the quantity of hyperoside. The main phenolic acid is chlorogenic acid, also being in a relatively constant concentration. Significant differences dependent on the development stage were observed for catechin and free quercetin. The catechin’s content dropped during the buds’ development. The results of LC/MS analyses are concordant with the spectral analyses’ results.

### 3.2. Corylus avellana Gemmotherapy Extract Improves Liver Function and Architecture of Fibrotic Livers in Diabetic Mice

**Figure 1:**
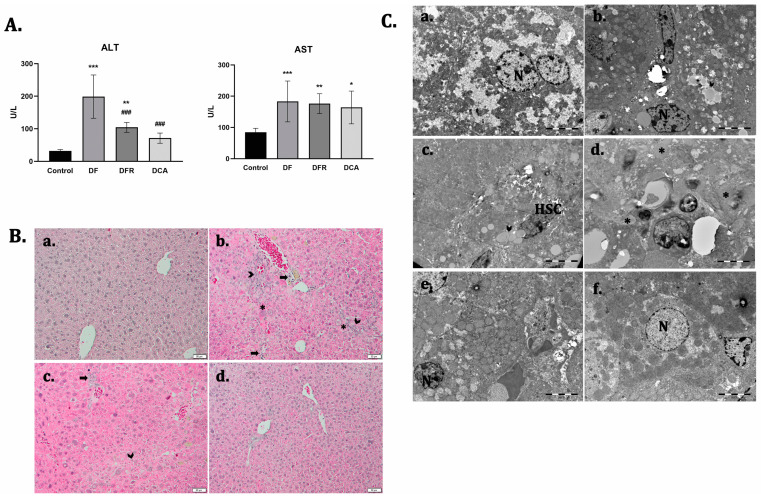
*Corylus avellana* gemmotherapy extract improves biochemical parameters and hepatic structure and ultrastructure of the fibrosis livers in diabetic mice. (**A**) Biochemical parameters of aspartate-amino-transferase (AST); alanine-amino-transferase (ALT); Values are expressed as mean ± SD (n = 10). *** *p* < 0.001 compared to control; ** *p* < 0.01 compared to control; * *p* < 0.05 compared to control; ^###^ *p* < 0.001 compared to DF; Legend: Control; *DF*—liver fibrosis in diabetic mice; *DFR*—self-recovery of liver fibrosis (positive control) in diabetic mice; *DCA*—*Corylus avellana* treatment of liver fibrosis in diabetic mice. (**B**) Representative light microscopy micrographs of liver histology in H&E stain, (**a**) control; (**b**) DF; (**c**) DFR, (**d**) DCA, Scale bar: 50 μm; Fibrosis (arrowhead), foam hepatocyte (*), inflammatory infiltrate (arrow). (**C**) Representative electron microscopy micrographs of the liver for the experimental groups; (**a**) control; (**b**–**d**) DF; (**e**) DFR; (**f**) DCA. N—hepatocyte’s nuclei; HSC—hepatocyte stellate cells; lipids (arrowhead); collagen (*); glycogen (G).

To evaluate the anti-fibrotic potential of the *Corylus avellana* gemmotherapy extract to alleviate liver fibrosis in a diabetic mouse model, we injected carbon tetrachloride for 7 consecutive weeks. Fibrotic mice showed a significant increase in the serum aspartate aminotransferase (AST) and alanine aminotransferase (ALT) compared to the control. However, a decrease in the serum levels of both AST and ALT was observed after 2 weeks of extract administration, compared to the fibrotic group (Figure 1A).

The histological analysis of the control livers revealed a regular lobular architecture with a centrilobular vein and radiating cords of hepatocytes (Figure 1B). Liver samples from the fibrosis group showed severe changes in morphology, obvious collagen deposition, pseudolobule formation and inflammatory cell infiltration in the interstitial areas. Numerous hepatocytes with micro- and macro-vesicular steatosis and sinusoid capillary congestion were also observed. The presence of hypertrophied perivenular “foamy hepatocytes” with a massive accumulation of microvesicular fat was noticed.

Moreover, electron microscopy analysis of the fibrotic livers revealed the presence of activated hepatic stellate cells and a massive accumulation of lipids into foam cells, a smooth endoplasmic reticulum proliferation. Also, bundle of collagen fibers that proliferated into the parenchyma, glycogen depletion and inflammatory cells that infiltrated into the parenchyma were observed (Figure 1C).

After two weeks of de novo recovery, in the absence of treatment, the significant presence of fibrous tissue and pseudo-lobules, accompanied by inflammatory cell infiltrates, was observed, compared to the fibrotic group. The ultrastructural alterations were observed in the self-recovery group, similar to fibrotic livers. After treatment with *Corylus avellana* extract, both the hepatic structure and ultrastructure were significantly improved compared to both the fibrotic and self-recovery groups.

### 3.3. Corylus avellana Gemmotherapy Buds Extract Suppresses the Secretion and Deposition of Collagen in a Liver Fibrosis Model of Diabetic Mice

**Figure 2:**
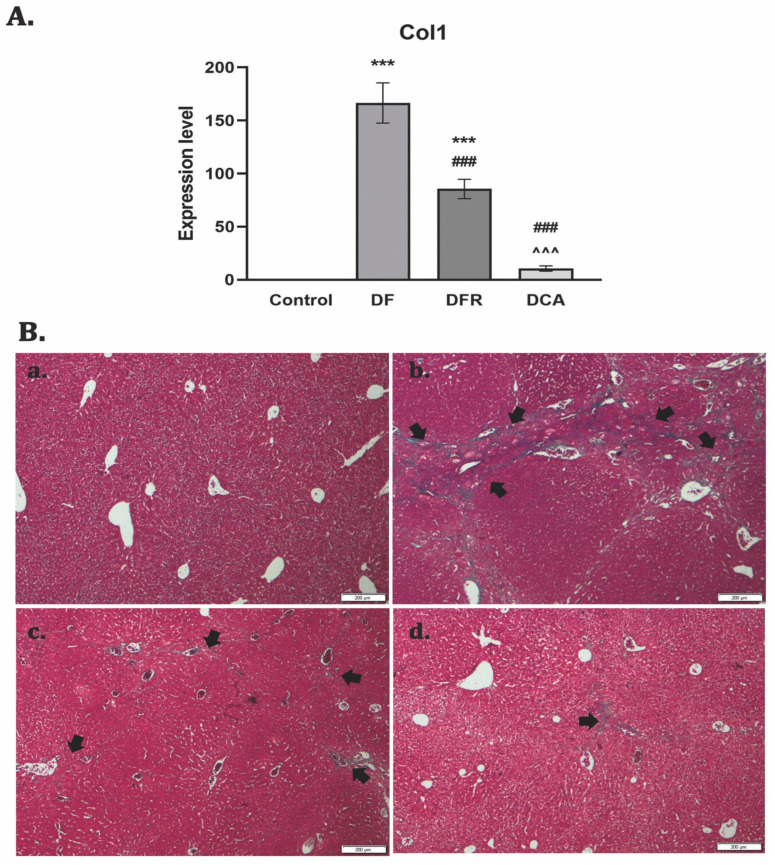
The effect induced by *Corylus avellana* gemmotherapy extract on reduction of collagen deposition in fibrotic livers of diabetic mice. (**A**) RT–PCR analysis of collagen 1 (Col 1) gene levels. Legend: Control, *DF*—liver fibrosis in diabetic mice; *DFR*—self-recovery of liver fibrosis (positive control) in diabetic mice; *DCA—Corylus avellana* treatment of liver fibrosis in diabetic mice; *** *p* < 0.001 compared to control; ^###^ *p* < 0.001 compared to *DF*; ^^^ *p* < 0.001 compared to *DFR*; (**B**) Collagen staining with Gomori’s trichrome kit. (**a**) Control group: no significant collagen deposition; (**b**) *DF* group: significant collagen deposition with pseudo-lobular separation (arrows); (**c**) *DFR* group: aspect almost similar to the LF-DIA group (arrows); (**d**) *DCA* less collagen deposition (isolated islands) compared to *DF* and *DFR* (arrows).

The RT–PCR analysis showed significant up-regulation of the Col-1 gene expression for the fibrotic group (*p* < 0.001). After two weeks of CA extract administration, a significantly decreased level of gene expression compared to the fibrotic group was noticed. (Figure 2A).

Collagen proliferation and deposition were observed using Gomori’s trichrome stain kit (Figure 2B). The control liver had a normal lobular structure without any proliferation of collagen, while diabetic mice with liver fibrosis showed significant collagen deposition and formation of pseudo-lobules. The extent of fibrotic change was still noticed after two weeks of toxic cessation. Treatment with the extract reduced the thickness and expansion of the fibrous septa to isolated collagen islands.

### 3.4. Corylus avellana Gemmotherapy Extract Inhibits Activation and Proliferation of HSCs in Fibrotic Livers of Diabetic Mice

**Figure 3:**
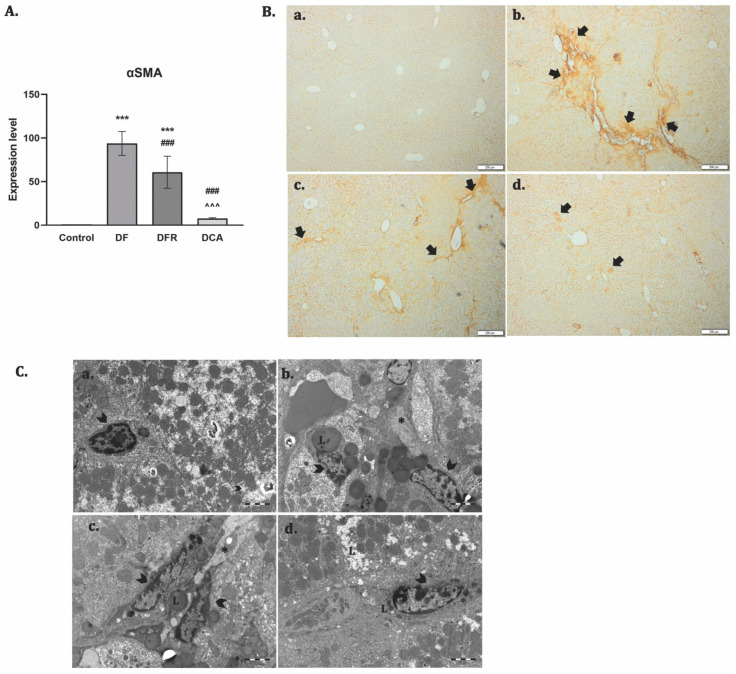
*Corylus avellana* gemmotherapy extract *inhibits* activation and proliferation of HSCs. (**A**) RT–PCR analysis of α-SMA gene level. Legend: Control, *DF*—liver fibrosis in diabetic mice; *DFR*—self-recovery of liver fibrosis (positive control) in diabetic mice; *DCA*—*Corylus avellana* treatment of liver fibrosis in diabetic mice; *** *p* < 0.001 compared to control; ^###^ *p* < 0.001 compared to DF; ^^^ *p* < 0.001 compared to DFR; (**B**) Immunohistochemical expression of α-SMA in experimental livers: (**a**) Control; (**b**) *DF*—liver fibrosis in diabetic mice—strong α-SMA immunopositivity; (**c**) *DFR*—self-recovery of liver fibrosis (positive control) in diabetic mice—α-SMA immunopositivity almost similar to DF group; (**d**) *DCA*—*Corylus avellana* treatment of liver fibrosis in diabetic mice; Scale bar: 200 μm. (**C**) Electron microscopy micrographs of the hepatic stellate cells; Legend: (**a**) Control; (**b**) *DF*—liver fibrosis in diabetic mice; (**c**) *DFR*—self-recovery of liver fibrosis (positive control) in diabetic mice; (**d**) *DCA*—*Corylus avellana* treatment of liver fibrosis in diabetic mice; hepatic stellate cells (arrowhead), lipids (L), collagen (*).

The expression of α-SMA shows the activated status of the hepatic stellate cells (HSCs). The RT–PCR analysis showed significant up-regulation of the α-SMA gene expression for the fibrotic group (*p* < 0.001). The DCA group presented significant down-regulation compared to the DF group (Figure 3A). The immunohistochemical analysis showed positive staining for the slides of fibrotic livers and a reduced expression almost to the level of control after the treatment, while de novo recovery of the liver (DFR) was close to the fibrotic one (DF) (Figure 3B). Activation and proliferation of the HSCs and the accumulation of the parenchymal lipids were observed by TEM for the fibrotic group, while DCA liver grids were depleted of lipids and with HSCs with a quiescent phenotype, almost similar to the control (Figure 3C).

### 3.5. Corylus avellana Gemmotherapy Extract Downregulates TGF-β1/Smad Signaling in Fibrotic Livers of Diabetic Mice

**Figure 4:**
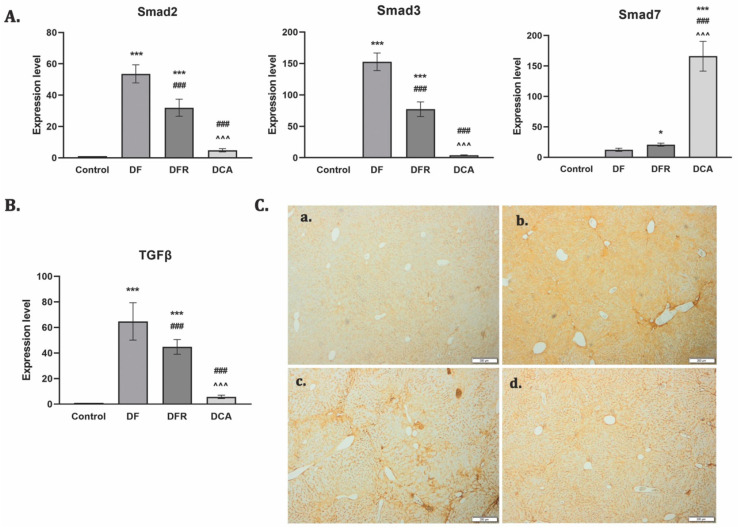
*Corylus avellana* gemmotherapy extract inhibits pro-fibrotic TGF-β1/Smad signaling. (**A**) RT–PCR analysis of Smad 2, 3 and 7 gene levels. Legend: Control; *DF*—liver fibrosis in diabetic mice; *DFR*—self-recovery of liver fibrosis (positive control) in diabetic mice; *DCA*—*Corylus avellana* treatment of liver fibrosis in diabetic mice; *** *p* < 0.001 compared to control; * *p* < 0.05 compared to control; ^###^ *p* < 0.001 compared to DF; ^^^ *p* < 0.001 compared to DFR; (**B**) RT–PCR analysis of TGF-β1 gene level. Legend: Control; *DF*—liver fibrosis in diabetic mice; *DFR*—self-recovery of liver fibrosis (positive control) in diabetic mice; *DCA*—*Corylus avellana* treatment of liver fibrosis in diabetic mice; *** *p* < 0.001 compared to control; ^###^ *p* < 0.001 compared to DF; ^^^ *p* < 0.001 compared to DFR; (**C**) Immunohistochemical expression of TGF-β1 in experimental livers. Legend: (**a**) Control; (**b**) *DF*—liver fibrosis in diabetic mice; (**c**) *DFR*—self-recovery of liver fibrosis (positive control) in diabetic mice; (**d**) *DCA*—*Corylus avellana* treatment of liver fibrosis in diabetic mice.

TGF-β1 is considered a key player in fibrogenesis through Smad 2/3 phosphorylation, while Smad 7 induced its inhibition. RT–PCR analysis showed an upregulation of the TGF-β1 gene expression and a strong immunopositivity for the DF group. The TGF-β1 gene level was significantly reduced by extract administration (*p* < 0.001) (Figure 4B). The same pattern was obtained for Smad2. In contrast, 2 weeks of *Corylus avellana* significantly upregulated Smad7 compared to the fibrotic group (Figure 4A).

### 3.6. Corylus avellana Gemmotherapy Extract Modulates ECM by TIMP-1/MMPs Balance

**Figure 5:**
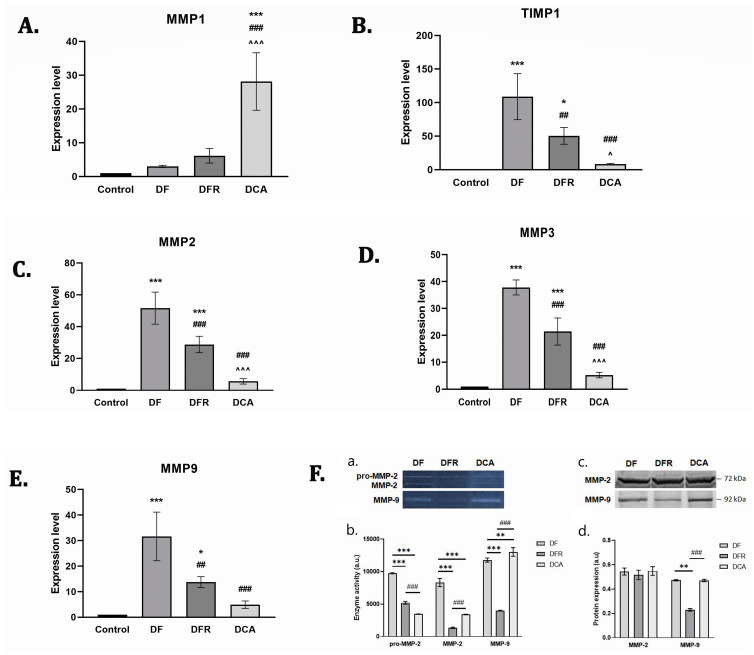
Effects of *Corylus avellana* gemmotherapy buds extract on tissue inhibitors of metalloproteinase (TIMP)-1/matrix metalloproteinase (MMP) pathway regulation. RT–PCR analysis of MMP1 (**A**), Timp-1 (**B**), MMP2 (**C**), MMP3 (**D**), MMP9 (**E**) gene expression levels. (**F**) Effects of on MMPs expression and enzymatic activity. (**a**) Images of gelatin zymography (pro-MMP-2, MMP-2, and MMP-9); (**b**) enzymatic activity of the metalloproteinases pro-MMP-2, MMP-2, and MMP-9, as well as (**c**) profile of MMP-2 and MMP-9 blots; (**d**) the expression of metalloproteinases MMP-2 and MMP-9 obtained following in vivo treatment of CA gemmotherapy extract. Legend: Control; *DF*—liver fibrosis in diabetic mice; *DFR*—self-recovery of liver fibrosis (positive control) in diabetic mice; *DCA—Corylus avellana* treatment of liver fibrosis in diabetic mice; *** *p* < 0.001 compared to control; * *p* < 0.05 compared to control; ** *p* < 0.01 compared to control; ^###^ *p* < 0.001; ^##^ *p* < 0.01; ^^^ *p* < 0.001 compared to *DFR*; ^ *p* < 0.05 compared to *DFR*.

Liver ECM deposition is especially balanced by TIMP-1, which is an inhibitor of the MMPs degradation of matrix components. To investigate the inhibitory effects of Corylus avellana gemmotherapy extract on ECM deposition in fibrotic livers, the mRNA levels of TIMP-1 and MMP-1, 2, 3, and 9 were measured by RT-PCR analysis and the enzymatic activity of MMP-2 and MMP-9 and profiles were assessed by zymography and profiles by western blot. Our data showed that the expression levels of these genes were considerably increased (*p* < 0.001) in the diabetes group compared with the control. Treatment with the gemmotherapy extract considerably downregulated the mRNA levels of MMP-2, MMP-3, MMP-9, and TIMP-1 compared with both the DF and DFR control groups (*p* < 0.001). In contrast, the mRNA expression of MMP-1 was considerably higher after the treatment compared to those of the DF and DFR control groups (*p* < 0.001) (Figure 5).

### 3.7. Oxidative Stress Biomarkers

In this study, a series of biomarkers of oxidative stress was analyzed, such as the level of lipid peroxidation, the concentration of reduced glutathione, the products of advanced oxidation of proteins, the expression level of the protein Nrf2 and the Heme oxygenase-1.

**Figure 6:**
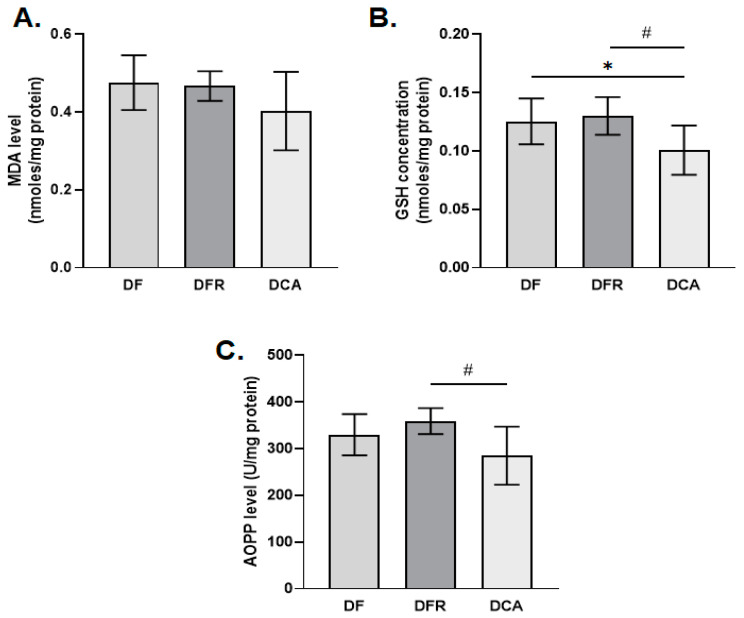
Effects of *Corylus avellana* (CA) extract on oxidative stress parameters. (**A**) The level of malondialdehyde (MDA), (**B**) the concentration of reduced glutathione (GSH), and (**C**) the products of advanced oxidation of proteins (AOPP) obtained after the in vivo administration of *Corylus avellana* (DCA) extract; *DF*—liver fibrosis in diabetic mice; *DFR*—self-recovery of liver fibrosis (positive control) in diabetic mice; *DCA—Corylus avellana* treatment of liver fibrosis in diabetic mice. # *p* < 0.05; (*)—group versus DF group; (#)—group versus DFR group.

**Figure 7:**
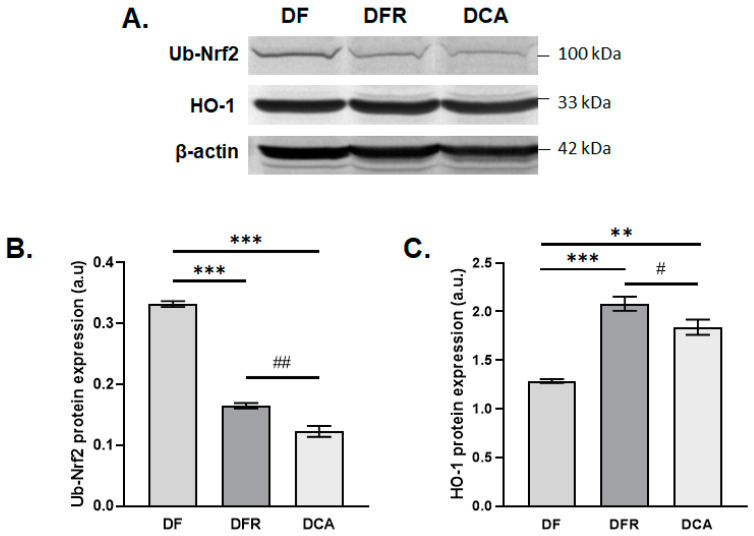
Effects of *Corylus avellana* (CA) extract on Ub-Nrf2 and HO-1 protein expression. (**A**) Protein bands on blot membrane; (**B**) expression of ubiquitinylated Nrf2 (Ub-Nrf2) protein, and (**C**) expression of heme oxygenase (HO-1) proteins after in vivo treatment of the *Corylus avellana* extract. DF–liver fibrosis in diabetic mice; DFR–self-recovery of liver fibrosis (positive control) in diabetic mice; DCA-*Corylus avellana* treatment of liver fibrosis in diabetic mice. ** *p* < 0.01; *** *p* < 0.001; ^##^ *p* < 0.01, ^#^ *p* < 0.05; (*)—group versus DF group; (#)—group versus DFR group.

The results obtained after the in vivo treatment showed that the level of lipid peroxidation slightly decreased in the CA group treated with the gemmotherapy extract compared to the group of fibrotic mice in an insignificant manner by about 1.2-fold compared to the DFR and DF samples. The concentrations of GSH and AOPP were significantly reduced in the DCA group compared to the DF and DFR ones by about 1.3-fold and 1.2-fold, respectively (Figure 6). The same pattern was for expression level of the protein Nrf2 was noticed (Figure 7).

## 4. Discussion

In this study, we set out to explore the hepatoprotective and liver fibrosis reversal effects of the gemmotherapy bud extract of *Corylus avellana*, because until now there are no scientific data to support this hypothesis. Following the treatment with *Corylus avellana*, we observed a reduction in fibrotic septa and collagen production, which may be due to the high content of polyphenols and especially hyperoside, which was identified in high concentrations regardless of the period harvesting buds. Studies have shown that hyperoside has an anti-inflammatory and antioxidant profile that may help to protect the liver from damage and slow the progression of liver fibrosis, by suppressing canonical NF-κB signaling [16], via upregulation of Nrf2 [17], or via the PHLPP2-AKT-GSK-3β signaling pathway [18].

Phytochemical analysis of the gemmotherapy buds extract of *Corylus avellana* revealed a high content of chlorogenic acid, a phenolic acid compound that has proven liver-protective and antifibrotic activity in in vitro and in vivo studies [19,20,21].

Our data showed that the GSH concentration increased in DFR samples compared to the control, suggesting that GSH is involved in the self-recovery of liver fibrosis in diabetic mice.

Previous studies regarding *Corylus avellana* kernels revealed the presence of phenolic acids, flavonoids such as myricetin, catechins, quercetin, proantocyanidines and lignans, as well as diarylheptanoids such as curcumin [7]. But in DCA individuals, the GSH concentration decreased significantly versus the DF and DFR ones, probably due to catechins that increased the activities of glutathione peroxidases [22] and lignans that up-regulated glutathione-S-transferase [23] both enzymes having as a co-substrate GSH and being involved in the neutralization/excretion of lipid peroxides. On the other hand, it is probable that the antioxidant compounds of *Corylus avellana* could diminish the production of the end-products of lipid peroxidation and AOPPs by direct interaction with them and by reactive oxygen species.

Nrf2 is a basic leucine-zipper (bZIP) redox active transcription factor that regulates the expression of numerous cytoprotective genes involved in the antioxidant response and phase II detoxifying enzymes by binding to antioxidant response elements (AREs) located in the promoters of these genes. Nrf 2 ubiquitination leads to its degradation at the level of 26S proteasome, contributing to a low basal ARE activity [24]. Some studies revealed that curcumin [25] and quercetin promote the translocation of Nrf2 to the nucleus, up-regulating the transcription of target genes [26] and generating a good therapeutic response [27]. Some of these genes are those encoding for heme oxygenases, enzymes existing in two isoforms, one inducible (HO-1) and one constitutively expressed. Considering that hyperoside is the main flavonoid of the *Corylus avellana* gemmotherapy buds extract, it is important to highlight its protective effect on carbon tetrachloride-induced chronic liver fibrosis in mice via upregulation of Nrf2 [17].

Heme oxygenases (HO) are essential enzymes that break down heme into carbon monoxide (CO), biliverdin, and free iron possessing anti-inflammatory and anti-apoptotic properties [28].

The up-regulation of HO-1 could protect the liver against inflammation via its products, CO, and biliverdin [29]. Recent studies proved that HO-1 induction could inhibit hepatic stellate cell proliferation [30] and, as a result, the expression of α-SMA decreased, as was shown in our study too.

Matrix metalloproteinases (MMPs) are a family of zinc-dependent enzymes involved in the remodeling of the extracellular matrix in physiological and pathological conditions. There are more than 25 different MMPs in mammalian cells. Between them, important are MMP2 and MMP9, also known as gelatinase A and gelatinase B, respectively, having as principal substrates collagens IV and V, gelatin, elastin and laminin.

The proMMP2 is constitutively expressed at the hepatocyte surface and is activated mainly by MT1-MMP. Unlike this, pro MMP-9 is not constitutively expressed, being inducible, and its activation is mediated by a second MMP (such as MMP-2 or matrilysin) or plasmin. The main liver source of MMP-9 is represented by Kupffer cells [31]. In liver fibrosis, MMP-2 has a profibrogenic role and is highly expressed in myofibroblasts [32]. Both gelatinases are also implicated in the regeneration process during liver inflammation and fibrosis [33]. The activation of MMP-2 and MMP-9 could determine the activation of TGFβ, involved in the differentiation of quiescent stellate cells in myofibroblasts that produce type 1 collagen [34]. In our experiment, the decrease in MMP-2 and MMP-9 activity in DFR samples compared to the DF ones could be correlated with the increase in GSH concentration, as was proved previously [35,36]. It is probable that MMP2 and MMP9 activity increased in DFC samples compared to DFR ones, due to the presence of quercetin, which has such an effect on these enzymatic activities together with the induction of apoptosis of hepatic stellate cells [37]. On the other hand, there is evidence that curcumin up-regulated MMP2 and MMP-9, as well as MMP7 and MMP13 [38].

Due to the fact that gemmotherapy extracts have been poorly studied, a comparison with the literature is not possible. Moreover, the hazel is recognized more as an alimentary plant and less as a medicinal one. From the nutritional point of view, hazelnuts contain fatty acids, phytosterols, vitamins, polyphenols and amino acids. This phytochemical profile gives these nuts and their oil cholesterol-lowering and antioxidant effects [11,39]. We could not find studies that demonstrate the hepatoprotective effect by lowering the transaminases or the antifibrotic effect of the nuts or of hazelnut oil. The effects of hazel buds gemmotherapy extract demonstrated in this study can be compared with a Rosemary young shoots extract [40].

## 5. Conclusions

Our results suggest that *Corylus avellana* gemmotherapy extract may have anti-fibrotic effects and could be useful in the prevention and treatment of liver fibrosis. The hepatoprotective mechanism is complex and is based on HSC inhibition, a reduction in oxidative stress and liver damage, downregulation of the TGF-β1/Smad signaling pathway and MMP/TIMP rebalance.

## Figures and Tables

**Table 1:** Primer sequences for RT–PCR.

Target	Sense	Antisense
TGF-β1	5′ TTTGGAGCCTGGACACACAGTAC 3′	5′ TGTGTTGGTTGTAGAGGGCAAGGA 3′
α-SMA	5′ CCGACCGAATGCAGAAG GA 3′	5′ ACAGAGTATTTGCGCTCCGAA 3′
Smad 2	5′ GTTCCTGCCTTTGCTGAGAC 3′	5′ TCTCTTTGCCAGGAATGCTT 3′
Smad 3	5′ TGCTGGTGACTGGATAGCAG 3′	5′ CTCCTTGGAAGGTGCTGAAG 3′
Smad 7	5′ GCTCACGCACTCGGTGCTCA 3′	5′ CCAGGCTCCAGAAGAAGTTG 3′
Col I	5′ CAGCCGCTTCACCTACAGC 3′	5′ TTTTGTATTCAATCACTGTCTTGCC 3′
MMP1	5′ GCAGCGTCAAGTTTAACTGGAA 3′	5′ AACTACATTTAGGGGAGAGGTGT 3′
MMP2	5′ CAG GGA ATG AGT ACT GGG TCT ATT 3′	5′ ACT CCA GTT AAA GGC AGC ATC TAC 3′
MMP3	5′ ACCAACCTATTCCTGGTTGCTGCT 3′	5′ ATGGAAACGGGACAAGTCTGTGGA 3′
MMP9	5′ AAT CTC TTC TAG AGA CTG GGA AGG AG 3′	5′ AGC TGA TTG ACT AAA GTA GCT GGA 3′
Timp1	5′ GGTGTGCACAGTGTTTCCCTGTTT 3′	5′ TCCGTCCACAAACAGTGAGTGTCA 3′
GAPDH	5′ CGACTTCAACAGCAACTCCCACTCTTCC-3′	5′ TGGGTGGTCCAGGGTTTCTTACTCCTT 3′

**Table 2:** The results of spectral determinations.

*Corylus avellana* Gemmotherapy Buds Extract Period of Harvesting	Total Flavonoids Expressed in Quercetine, mg/mL	Total Polyphenols Expressed in Caffeic Acid, mg/mL
February 2019	4.1 ± 0.08	65.3 ± 0.67
March 2019	4.8 ± 0.11	68.6 ± 0.85
January 2020	4.1 ± 0.12	67.8 ± 0.51
February 2020	4.6 ± 0.10	70.6 ± 0.74

**Table 3:** Quantitative determinations of the fractions by LC/MS.

*Corylus avellana* Gemmotherapy Buds Extract/Standard	February 2019	March 2019	January 2020	February 2020
	mg/mL			
Chlorogenic acid	0.360 ± 0.0090	0.360 ± 0.0087	0.430 ± 0.0204	0.340 ± 0.0104
Gallic acid	0.080 ± 0.0024	0.070 ± 0.0021	0.070 ± 0.0022	0.080 ± 0.0030
Salicylic acid	<qL	0.040 ± 0.0009	0.070 ± 0.0031	0.060 ± 0.0018
Catechin	0.160 ± 0.0038	0.100 ± 0.0038	0.190 ± 0.0057	0.130 ± 0.0042
Apigenin	<qL	0.003 ± 0.0001	0.002 ± 0.0001	-
Chrysine	0.100 ± 0.0051	0.090 ± 0.0031	0.090 ± 0.0023	0.090 ± 0.0035
Quercetin	0.020 ± 0.0004	0.080 ± 0.0025	0.110 ± 0.0034	0.070 ± 0.0018
Hyperoside	2.000 ± 0.0371	2.000 ±0.0377	2.270 ± 0.0615	2.030 ± 0.0487
Rutoside	0.580 ± 0.0178	0.600 ± 0.0210	0.570 ± 0.0171	0.580 ± 0.0094
Luteolin-7-*O*-glucoside	0.070 ± 0.0027	0.070 ± 0.0018	0.070 ± 0.0009	0.070 ± 0.0026
Naringenin	0.020 ± 0.0007	0.020 ± 0.0008	0.020 ± 0.0005	0.030 ± 0.0008

## Data Availability

The data presented in this study are available on request from the corresponding author.

## Supplementary Materials

The following supporting information can be downloaded at: https://www.mdpi.com/article/10.3390/biomedicines11061771/s1, Figure S1: The LC/MS chromatograms of *Corylus avellana* gemmotherapy extract (February 2019); Figure S2 The LC/MS chromatograms of *Corylus avellana* gemmotherapy extract (March 2019); Figure S3: The LC/MS chromatograms of *Corylus avellana* gemmotherapy extract (January 2020); Figure S4: The LC/MS chromatograms of *Corylus avellana* gemmotherapy extract (February 2020); Figure S5: MS spectra of the identified compounds from *Corylus avellana* gemmotherapy extract (February 2019); Figure S6: MS spectra of the identified compounds from *Corylus avellana* gemmotherapy extract (March 2019); Figure S7: MS spectra of the identified compounds from *Corylus avellana* gemmotherapy extract (January 2020); Figure S8: MS spectra of the identified compounds from *Corylus avellana* gemmotherapy extract (February 2020); Figure S9: LC/MS chromatograms of the standards; Figure S10: MS spectra of the standards.
