# Supplementary material for: Phytochemical Profiling and Anti-Fibrotic Activities of the Gemmotherapy Bud Extract of Corylus avellana in a Model of Liver Fibrosis on Diabetic Mice
Source: Biomedicines. 2023 Jun 20;11(6):1771. doi: 10.3390/biomedicines11061771 (PMC10296144; doi:10.3390/biomedicines11061771)
Supplement: Supplementary file 1 [file biomedicines-11-01771-s001.zip › biomedicines-2421169-supplementary.pdf]

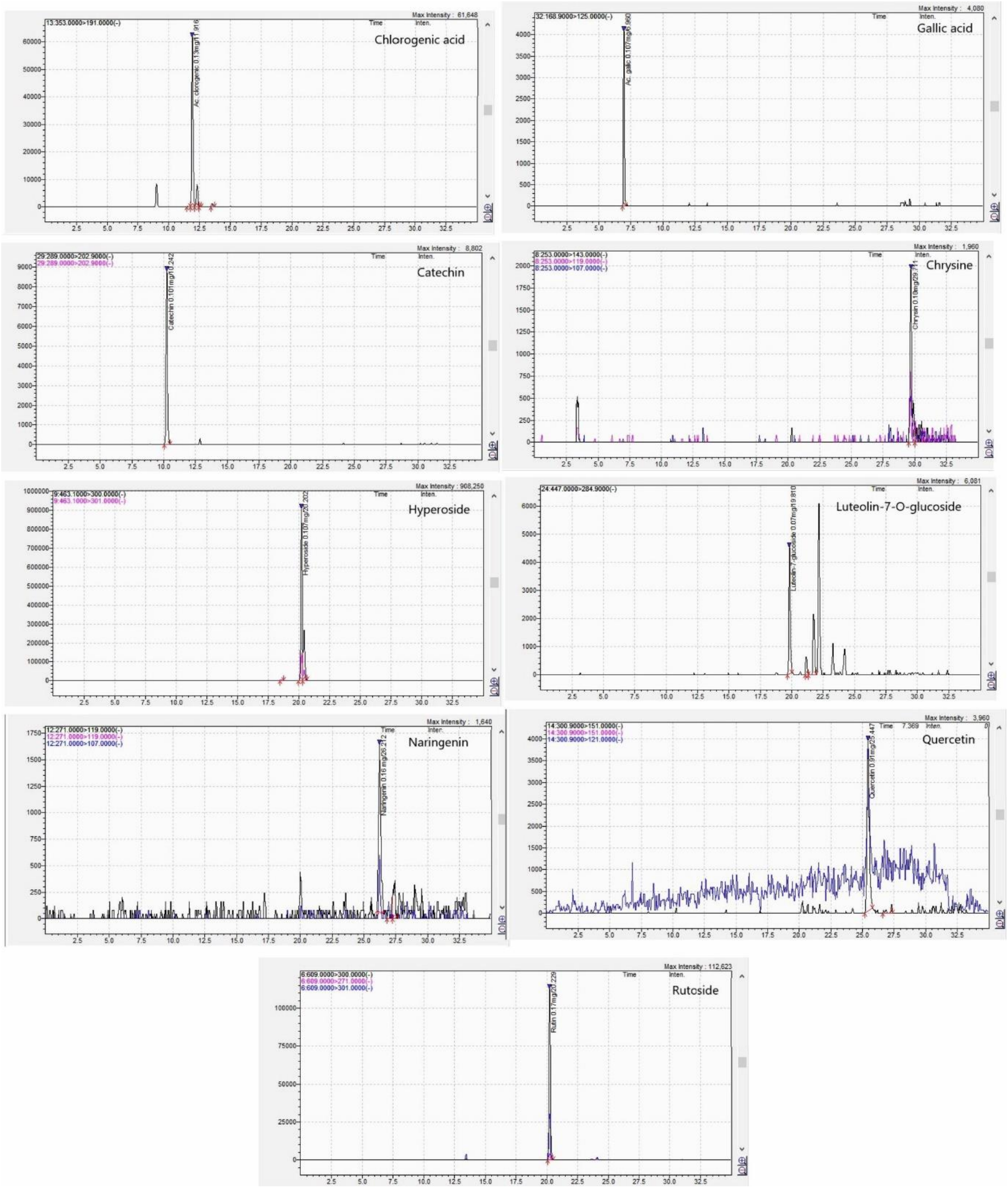

Figure S1: The LC/MS chromatograms of *Corylus avellana* gemmotherapy extract (Feb. 2019).

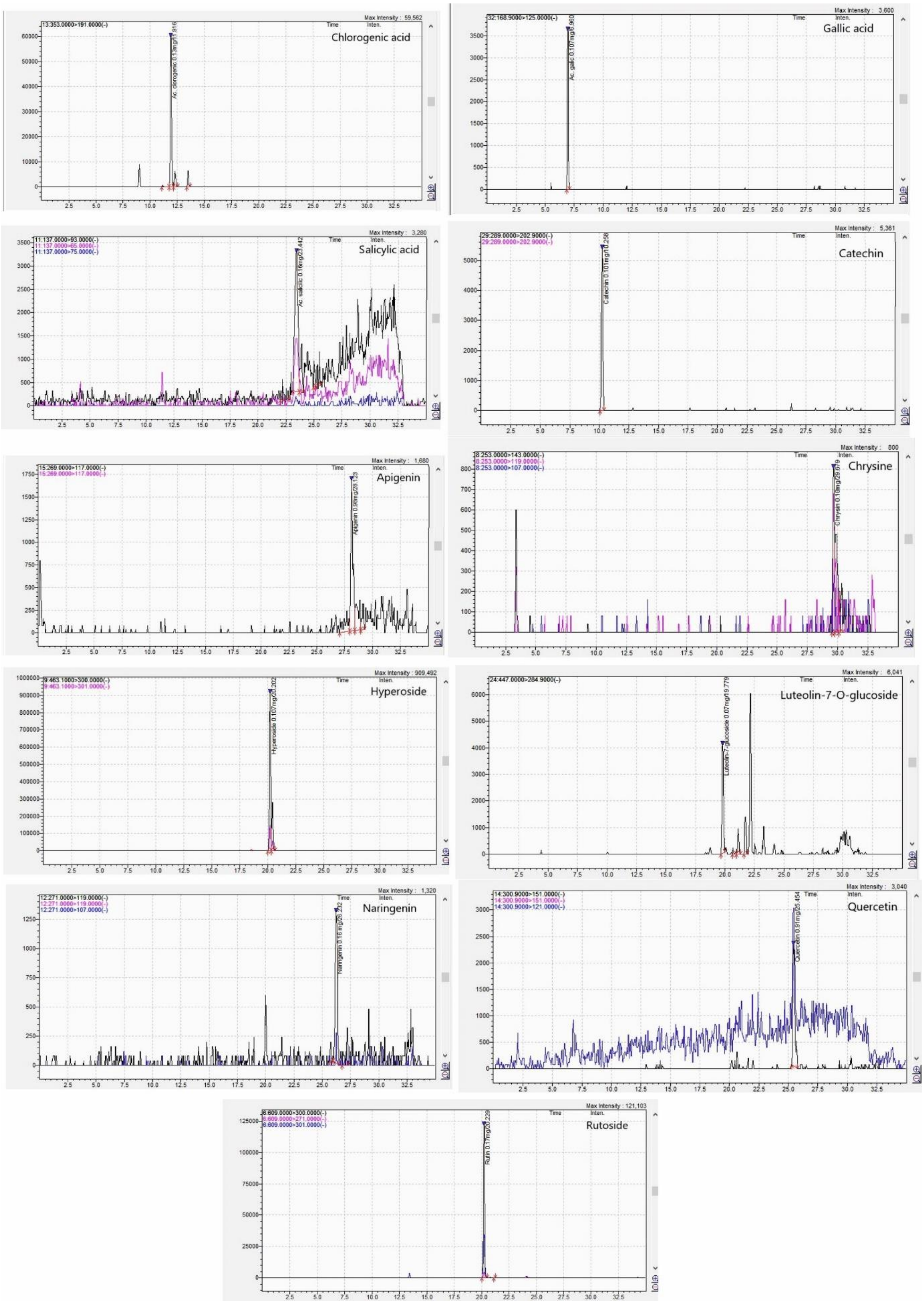

Figure S2: The LC/MS chromatograms of *Corylus avellana* gemmotherapy extract (Mar. 2019).

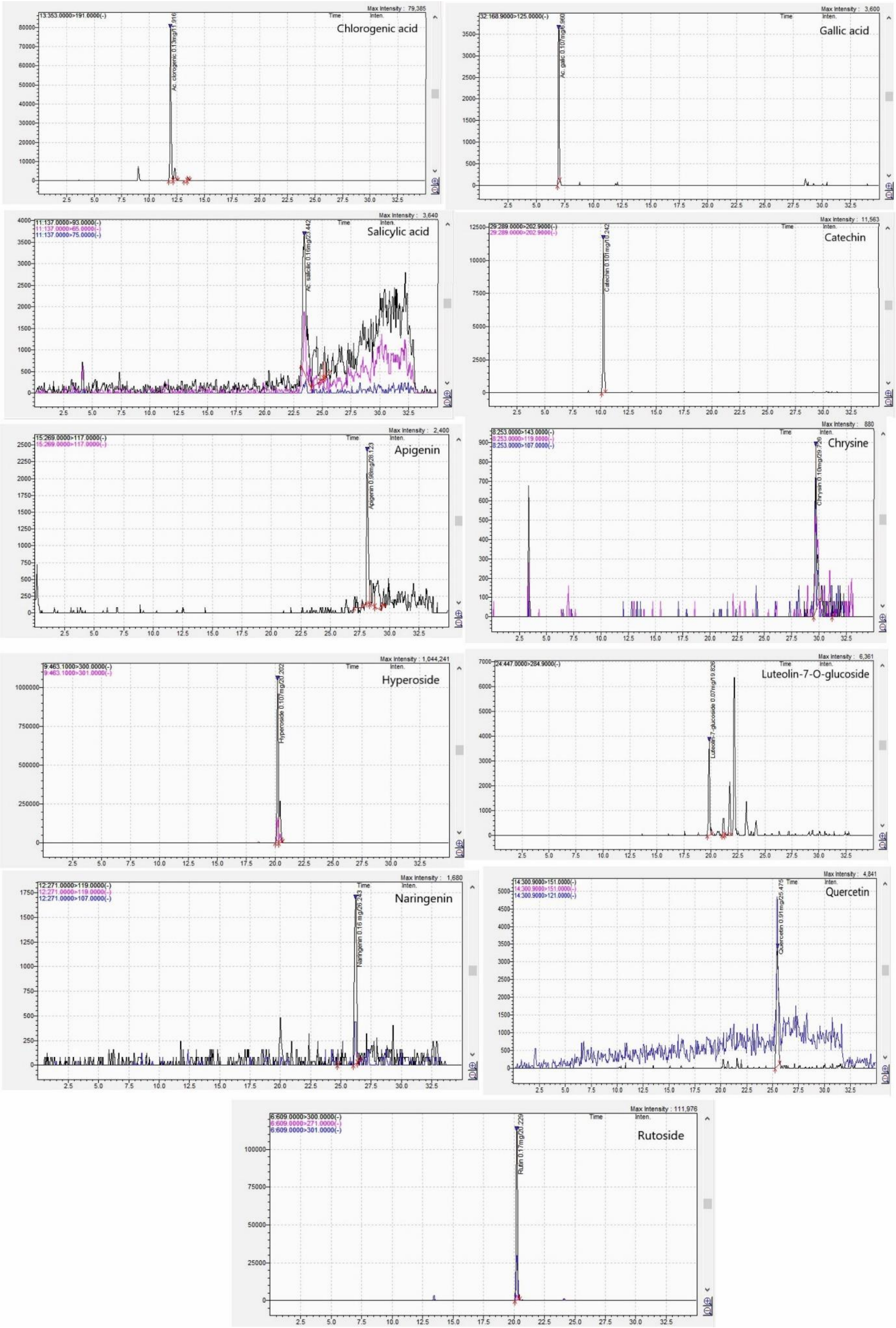

Figure S3: The LC/MS chromatograms of *Corylus avellana* gemmotherapy extract (Jan. 2020).

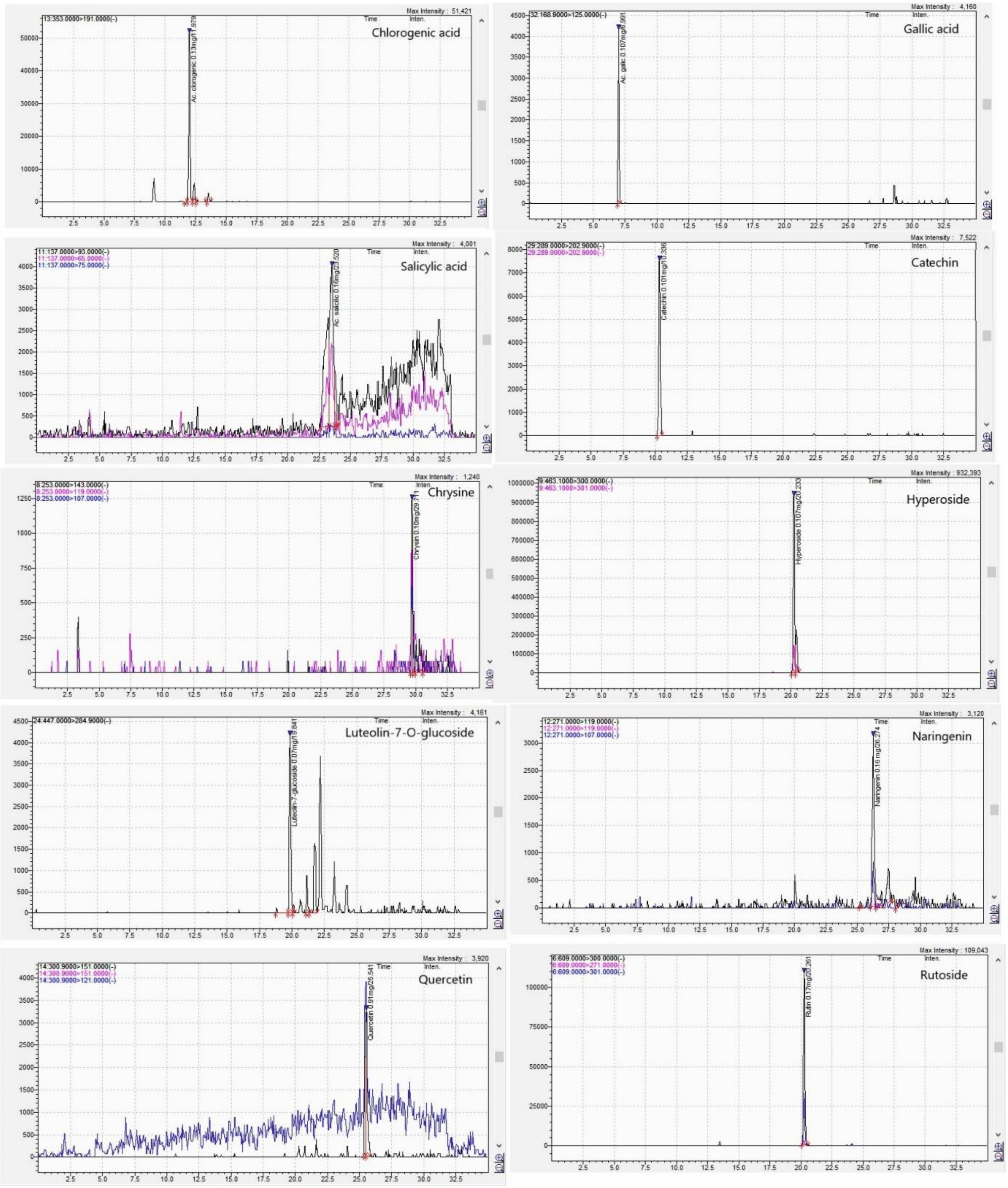

Figure S4: The LC/MS chromatograms of *Corylus avellana* gemmotherapy extract (Feb. 2020).

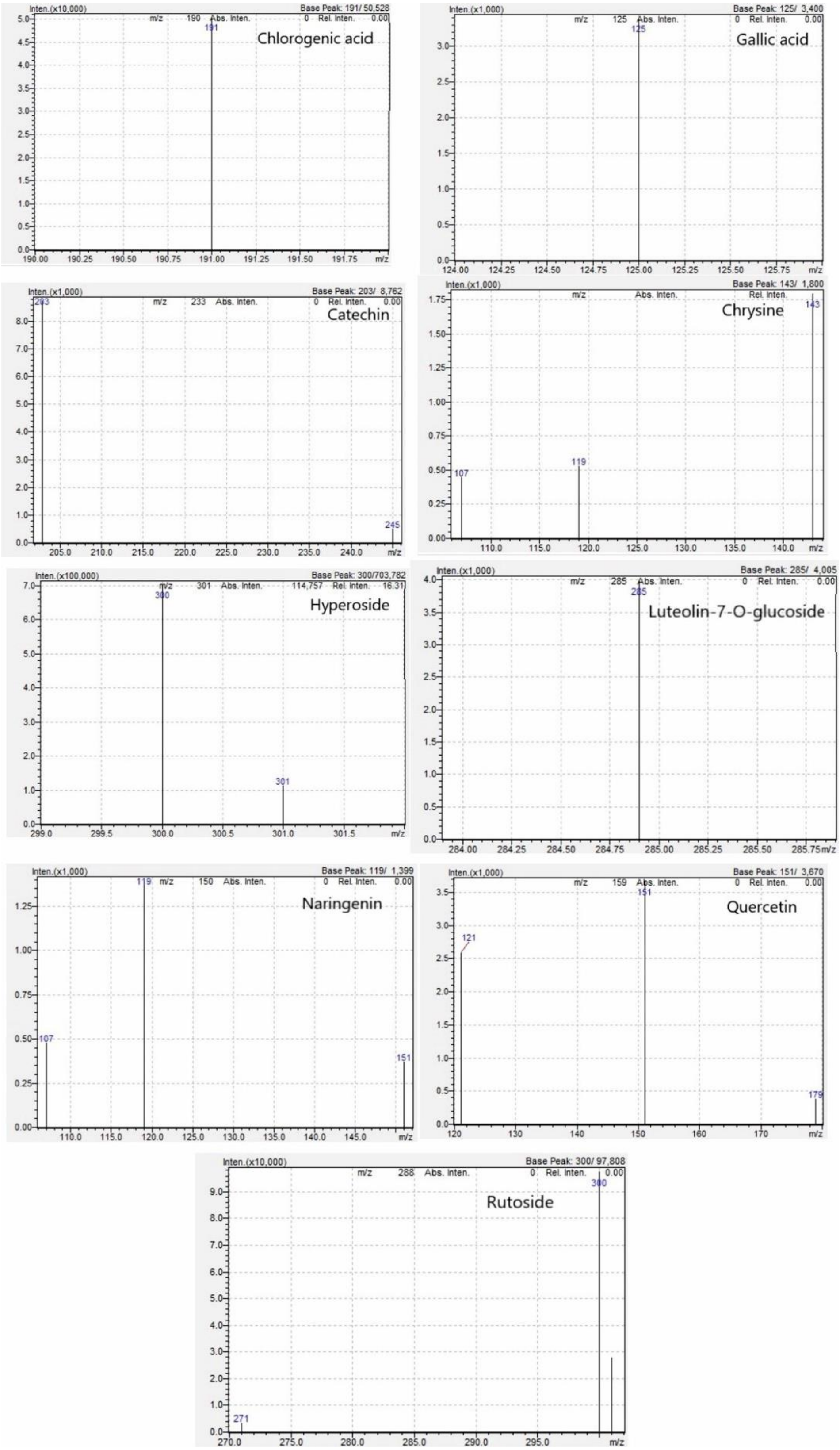

Figure S5: MS spectra of the identified compounds from *Corylus avellana* gemmotherapy extract (Feb. 2019).

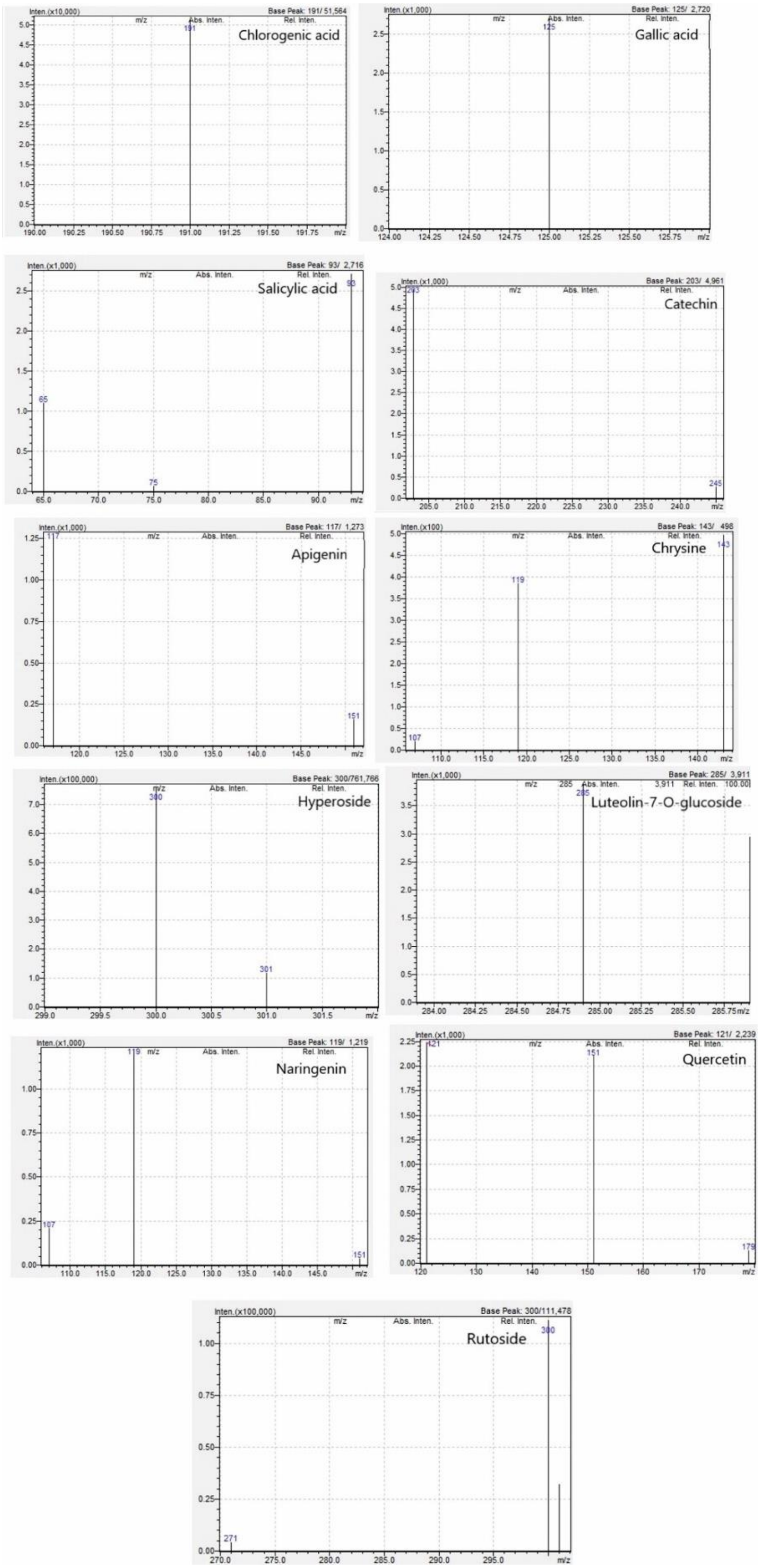

Figure S6: MS spectra of the identified compounds from *Corylus avellana* gemmotherapy extract (March 2019).

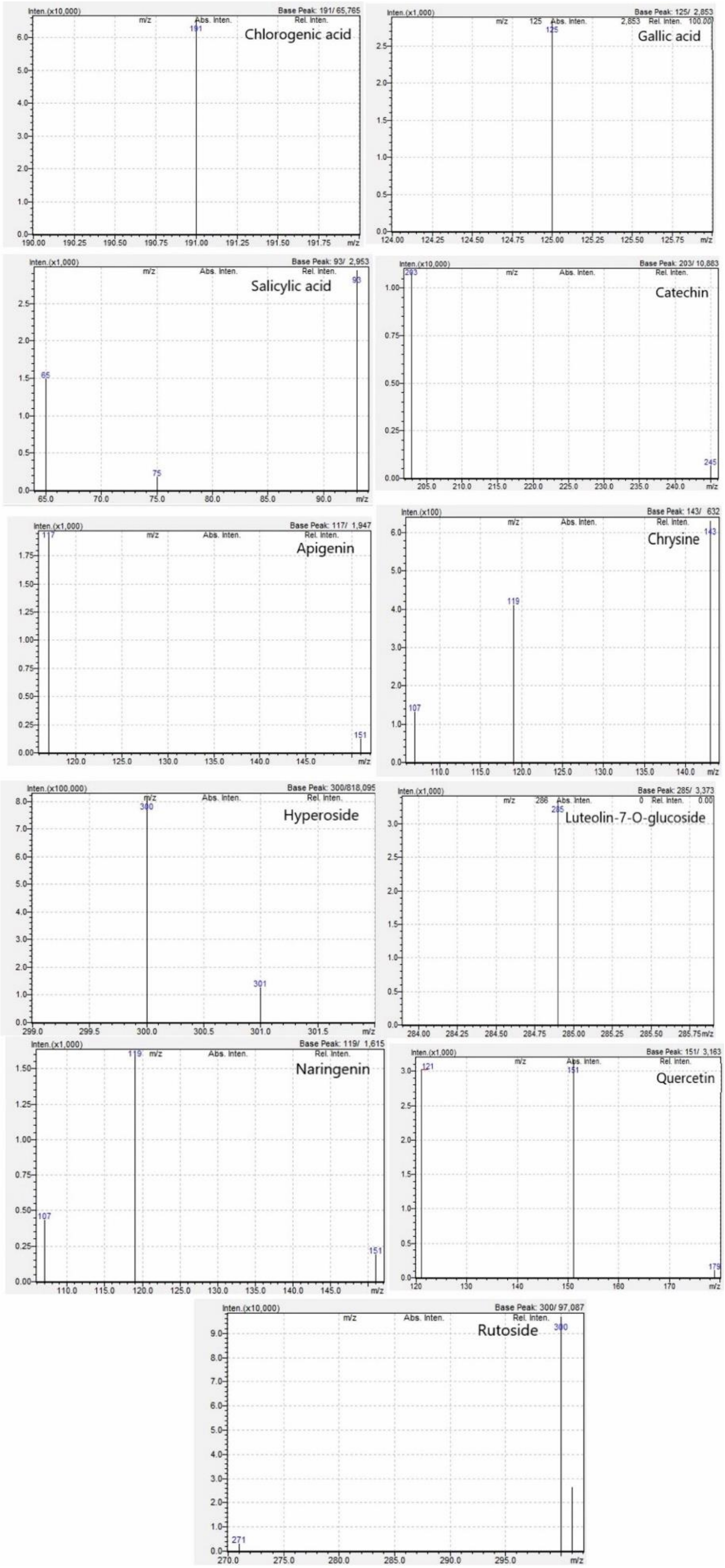

Figure S7: MS spectra of the identified compounds from *Corylus avellana* gemmotherapy extract (Jan. 2020).

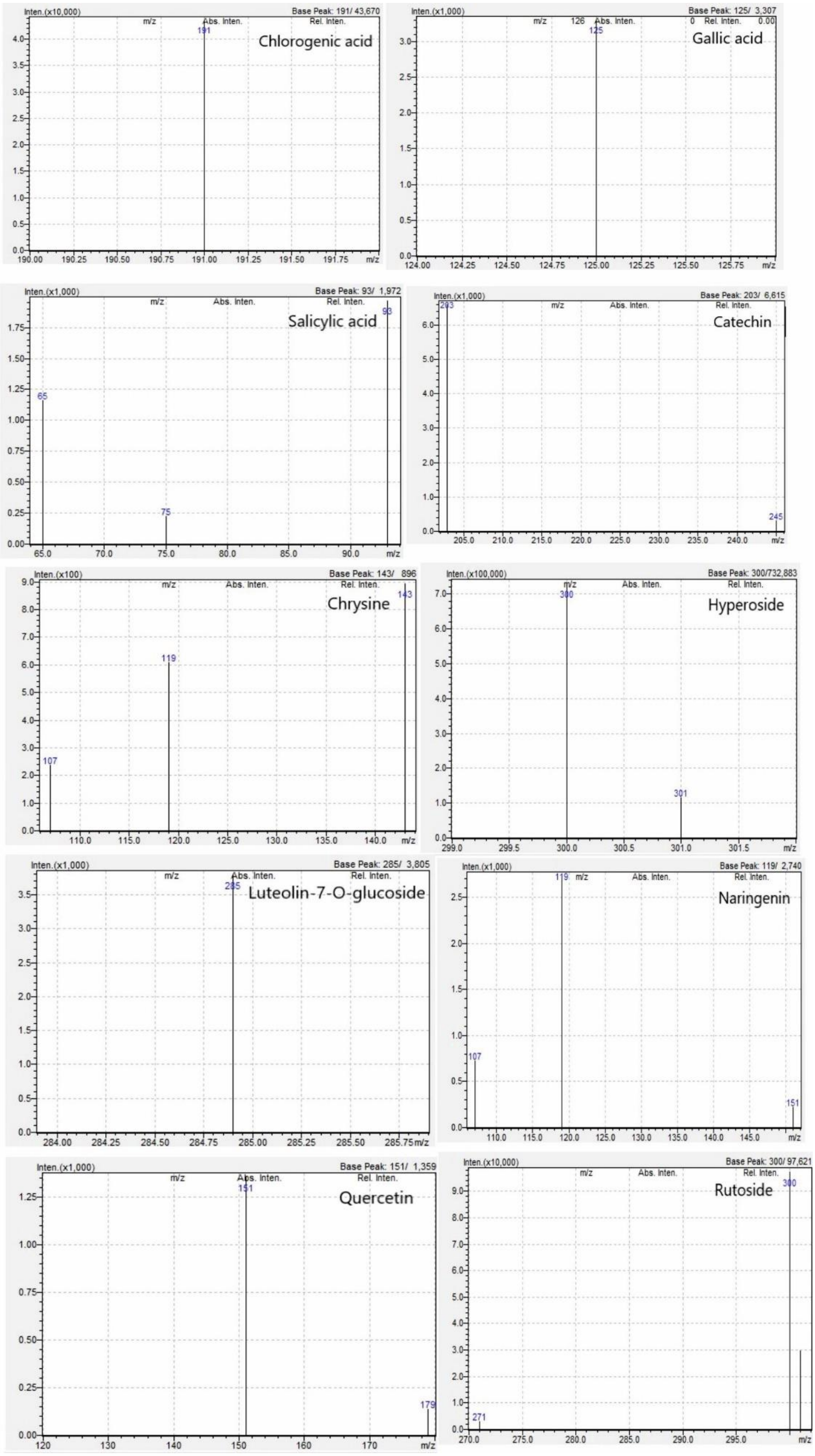

Figure S8: MS spectra of the identified compounds from *Corylus avellana* gemmotherapy extract (Feb. 2020).

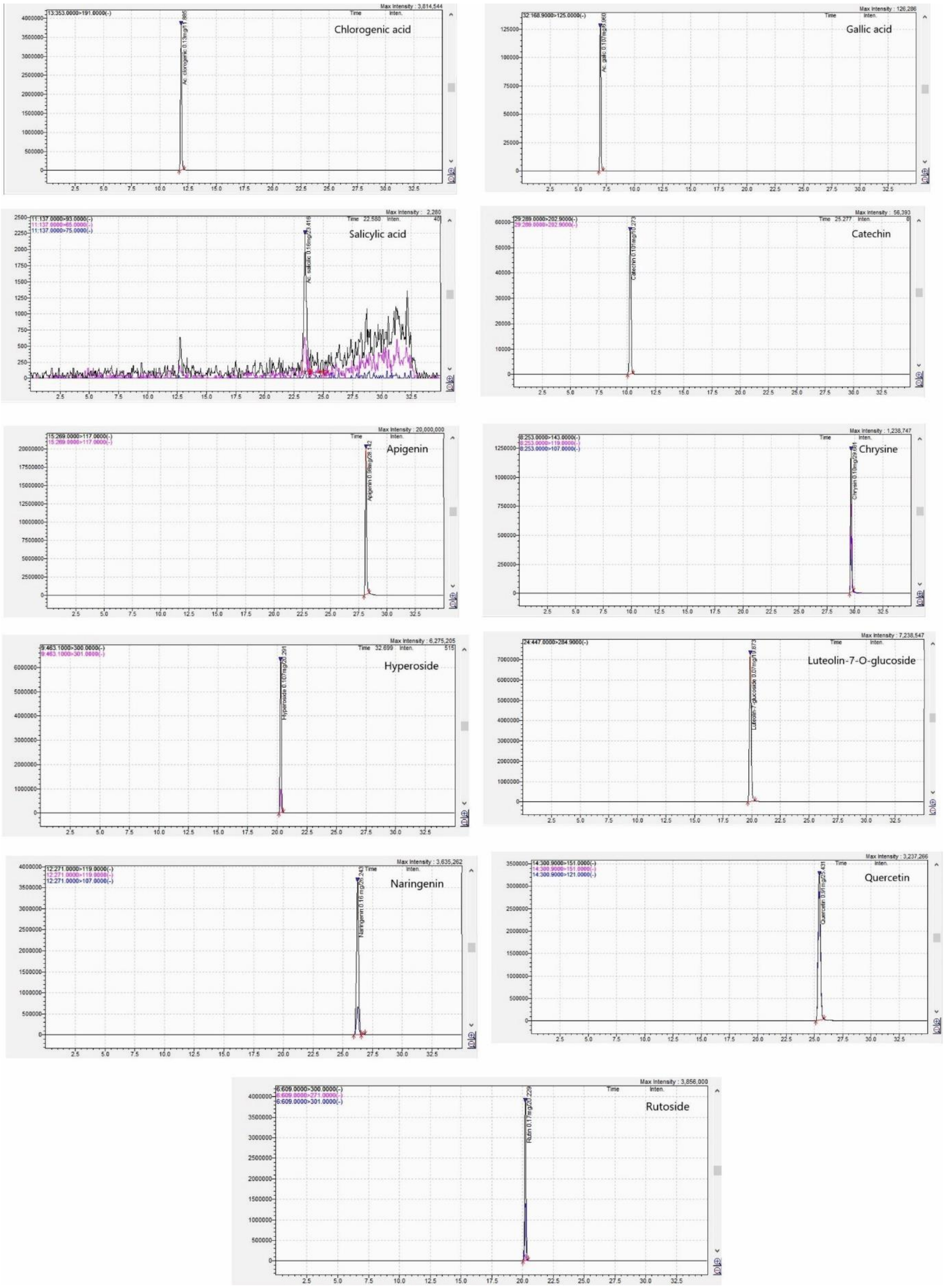

Figure S9: LC/MS chromatograms of the standards.

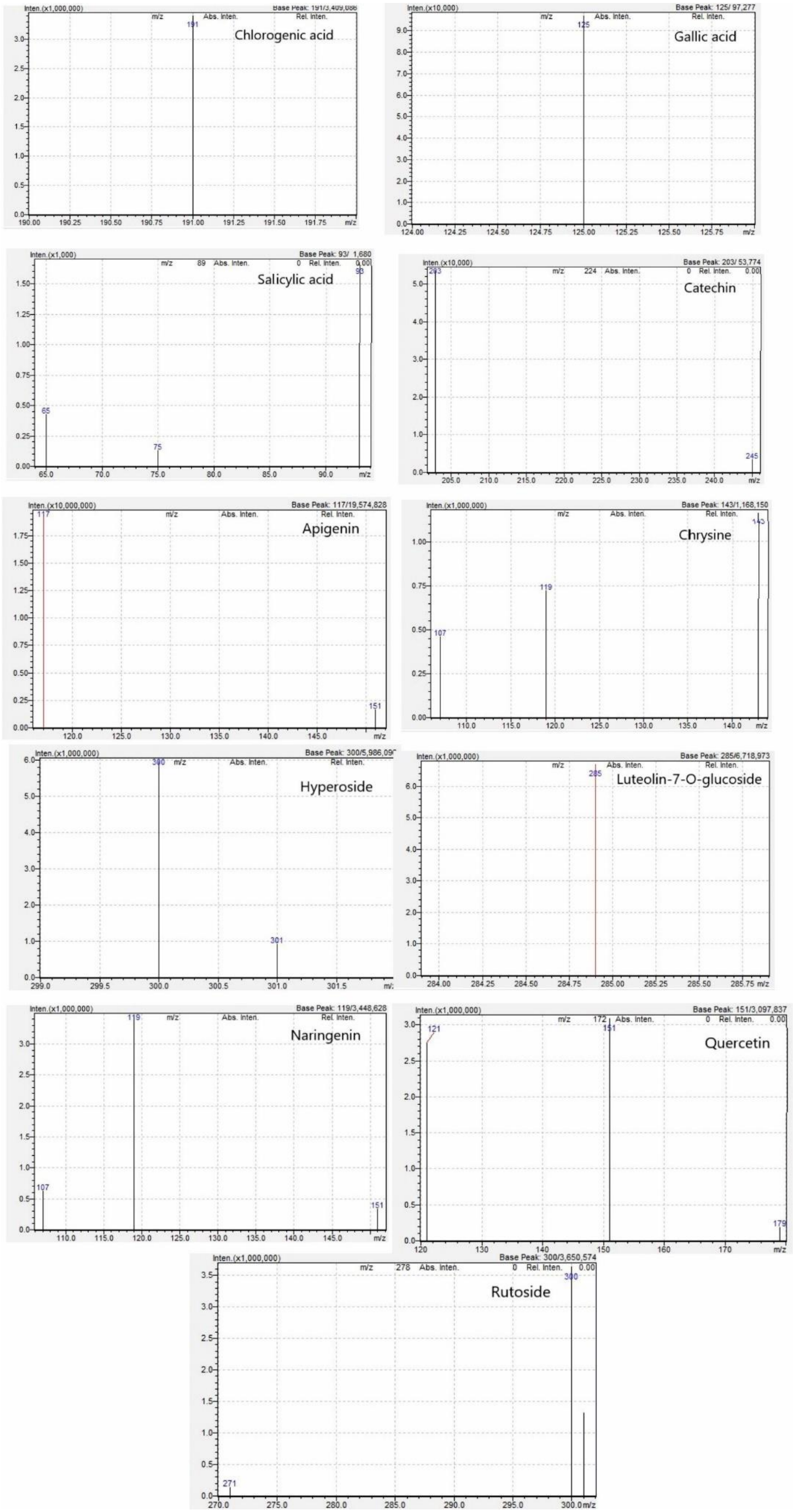

Figure S10: MS spectra of the standards.
